# Effect of La on the Microstructures and Mechanical Properties of Al-5.4Cu-0.7Mg-0.6Ag Alloys

**DOI:** 10.3390/ma17164141

**Published:** 2024-08-21

**Authors:** Xiang Li, Anmin Li, Xiangdu Qin, Hailong Yang, Peng Cheng

**Affiliations:** 1 School of Resources, Environment and Materials, Guangxi University, Nanning 530004, China; 15177617346@163.com (X.L.); 15078266480@163.com (X.Q.); 18743203440@163.com (H.Y.); pengcheng5815@163.com (P.C.); 2 State Key Laboratory of Featured Metal Materials and Life-Cycle Safety for Composite Structures, Guangxi University, Nanning 530004, China; 3 MOE Key Laboratory of New Processing Technology for Nonferrous Metals and Materials, Guangxi University, Nanning 530004, China

**Keywords:** cast Al-Cu-Mg-Ag, La, microstructure, high-temperature tensile mechanical property

## Abstract

The effects of the rare earth element La on the microstructure and mechanical properties of cast Al-5.4Cu-0.7Mg-0.6Ag alloys have been investigated through metallographic observation, scanning electron microscopy analysis, transmission electron microscopy, X-ray diffraction, and tensile testing. The present form and action mechanism of La have been analyzed. The findings indicate that the inclusion of trace amounts of La markedly diminishes the grain size in the Al-Cu-Mg-Ag alloy. Furthermore, as the La content increases, the alloy’s strength is significantly improved. When the La concentration reaches 0.4 wt.%, the mechanical properties of the alloy, both at room temperature and at 350 °C, surpass those of the alloy lacking rare earth elements. When the added rare earth La content exceeds 0.2 wt.%, the emergence of the Al_6_Cu_6_La phase causes the alloy structure to exhibit a skeletal morphology, altering the morphology and distribution of excess second phases along grain boundaries, thereby impacting the alloy’s overall performance. Incorporating La leads to a reduction in the size of the strengthening precipitate phase Ω while also enhancing its precipitation density, but an excess of La leads to the emergence of Al_6_Cu_6_La, depleting the available Cu and suppressing the precipitation of the Ω phase, ultimately affecting the mechanical properties of the alloy.

## 1. Introduction

Al-Cu alloys are lightweight alloys with excellent characteristics such as high strength, good toughness, and good corrosion resistance. They are rapidly being applied in the automotive, aerospace, and defense industries [1,2,3,4]. However, the casting performance of conventional Al-Cu alloys suffers from defects, and it is easy to form shrinkage voids and uneven second phases in the production process, resulting in premature cracks during tensile loading. Moreover, the enhancement of the alloy’s comprehensive performance by improving production processes remains limited. Therefore, the incorporation of minimal quantities of rare earth elements coupled with appropriate grain refinement and solution aging heat treatment methods can be employed to improve the microstructure of the alloy, thereby enhancing its mechanical properties [5,6,7].

Owing to the remarkable chemical reactivity of rare earth elements, they are capable of forming intermetallic compounds with other elements within the alloy matrix, promoting heterogeneous nucleation and refining α-Al dendrites [8]. Numerous investigations have been undertaken to refine the microstructure and enhance the mechanical properties of aluminum and magnesium alloys through the incorporation of rare earth elements like Ce, Yb, and Er [9,10,11]. The influence of minor La additions on the microstructure and mechanical properties of cast ADC12 alloys has been explored [12]. The results indicate that the refining effect on dendrites is most significant when the addition amount is 0.3 wt.%, and the alloy demonstrates optimal mechanical properties. However, when the La addition exceeds 0.6 wt.%, the formation of La-rich intermetallic compounds leads to a decline in the alloy’s property. Du et al. [13] explored the impact of incorporating trace amounts of La and Ce as composite modifiers on the microstructure and mechanical properties of cast Al-Cu-Mn-Mg-Fe alloy, and La and Ce had a substantially enhanced effect on the alloy, while the mechanical properties of the alloys with rare earth addition were superior to those of the matrix alloy without rare earth addition. Recent studies have found that adding 0.25% Ce and La to Al-Cu-Mn-Mg-Fe alloys also promotes the formation of denser T phases and finer AlMnFe phases, thereby increasing the tensile strength of the alloy [14]. Moreover, Wang et al. [15] studied the effect of adding rare earth Ce to Al-Cu-Mg-Ag alloys on their aging hardening and mechanical properties, discovering that Ce promotes the formation of the primary precipitate phase Ω and hinders the diffusion of Cu atoms in the precipitate phase at high temperatures, effectively enhancing the tensile strength at various temperatures. Some of the literature indicates that simultaneously adding Zr and Ce to Al-Cu alloys can significantly improve their high-temperature thermal stability. This is because Zr can enrich the Al matrix/Al_8_Cu_3_Ce interface, effectively inhibiting the phase transition from Al_8_Cu_3_Ce to Al_8_Cu_4_Ce at high temperatures [16]. Li et al. [8] studied the microstructure and mechanical properties of Al-5Cu alloys with added Sc, concluding that when the Sc content exceeds 0.4 wt.%, the Al_3_Sc phase refines the alloy grains and changes the morphology of the Al_2_Cu phase, significantly improving the tensile properties of the alloy.

However, there have been few reports on the influence of La on the microstructure and high-temperature mechanical properties of cast Al-5.4Cu-0.7Mg-0.6Ag alloy. Therefore, based on this background, this study further explores the effects of adding different compositions of La on the microstructure and mechanical properties of Al-Cu-Mg-Ag alloys and investigates the optimal composition and effect mechanism of rare earth La in Al-Cu-Mg-Ag alloys, providing a reference for the development of Al-Cu alloys with excellent high-temperature performance.

## 2. Materials and Methods

The experiment utilized industrial high-purity aluminum ingots alongside intermediate alloys of Al-50Cu, Al-20Mg, and Al-5Ag as raw materials for preparation. Firstly, pure aluminum ingots and graphite crucibles were placed together into a well-type resistance furnace and heated gradually. The temperature of the furnace was elevated to around 800 °C, and then, the required intermediate alloys were sequentially added. the covering agent was added and then stood and held for 10 min, followed by stirring and slagging. The power was adjusted, and when the furnace temperature dropped to around 680–700 °C, Mg was quickly injected into the bottom of the melt. After a 5 min insulation period for hexachloroethane refining treatment, the power was adjusted again to raise the furnace temperature to 710 °C for mechanical stirring for 2 min. When the furnace temperature reached 730–740 °C, slag removal was performed again. Finally, the temperature was maintained, and the crucible was taken out, and the alloy solution was poured into a preheated steel mold at around 200 °C. The Al-5.4Cu-0.7Mg-0.6Ag base alloy was melted in high-purity graphite crucibles according to a certain ratio (data before elements indicate mass fraction, %), and experimental alloys were prepared by adding 0.2, 0.4, and 0.6 wt.% La elements separately on this basis. The compositions of the alloys employed in the experiment are detailed in Table 1.

The ingots obtained by smelting were processed into samples and then subjected to T6 heat treatment. The alloys underwent solution treatment at 520 °C for 6 h, followed by rapid water quenching to room temperature. Finally, they were aged at 185 °C for 8 h and cooled in air. The four alloys were heat-treated simultaneously in the same furnace to ensure uniform heat treatment conditions.

As shown in Table 2, the actual compositions of the elements in the experimental alloys slightly deviated from the nominal compositions but remained within the permissible range. For accuracy and convenience, the compositions of the experimental alloys mentioned in this paper are based on the nominal compositions.

The alloy elements were quantitatively analyzed using an X-ray fluorescence spectrometer (S8 TIGER, BRUKER, Karlsruhe, Germany). The phase characterization of the alloy was performed utilizing an X-ray diffractometer (Rigaku D/MAX 2500V, Rigaku Corporation, Akishima, Japan). Room-temperature tensile properties of the alloy were tested on an universal testing machine (AGS-X 100KN, Shimadzu, Shanghai, China), featuring a gauge length of 25 mm and a strain rate of 0.5 mm/min. High-temperature mechanical properties were tested on a testing machine at a strain rate of 0.5 mm/min (Kappa, ZWICK, Ulm, Germany). The microstructures and second-phase particles of the alloys were examined using a field emission scanning electron microscope (Sigma 300, ZEISS, Oberkochen, Germany) with complementary EDS equipment for energy-dispersive X-ray spectroscopy to analyze the compositions of intermetallic compounds. Structural observations of the alloy were carried out using an FEI Tecnai G2 F20 transmission electron microscope (FEI Tecnai G2 F20, FEI, Hillsboro, OR, USA). For TEM analysis, samples were prepared using a twin-jet device in a mixed solution of nitric acid and methanol (volume ratio 3:7), with an applied voltage of 16 V at −30 °C. At least three samples of each alloy were tested to calculate the average values of strength and elongation.

## 3. Results

### 3.1. Microstructure of Alloys

**Figure 1:**
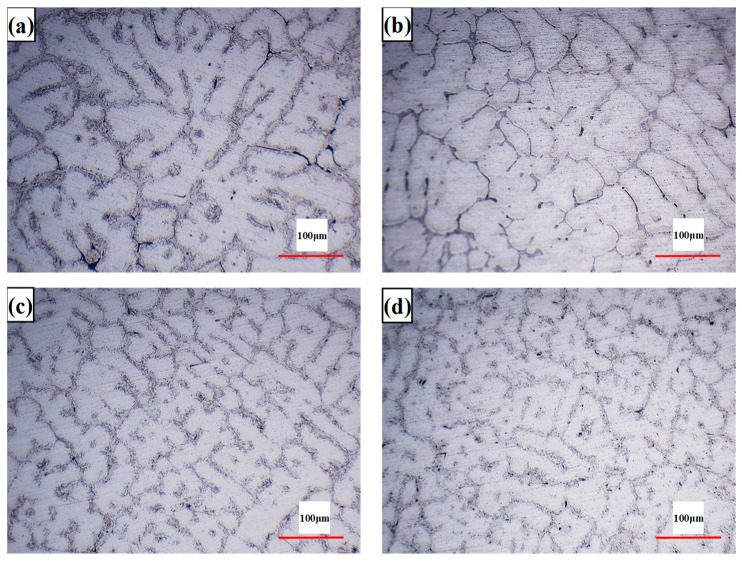
Optical micrographs of Al-5.4Cu-0.7Mg-0.6Ag-xLa alloy: (**a**) La-free; (**b**) 0.2La; (**c**) 0.4La; (**d**) 0.6La.

The metallographic microstructure of the Al-5.4Cu-0.7Mg-0.6Ag-xLa alloys is illustrated in Figure 1. As depicted, all alloys exhibit a typical dendritic microstructure. In the base alloy, which lacks the addition of rare earth element La, the grains mainly consist of coarse dendrites, with some equiaxed grains dispersed within, and numerous large undissolved second phases are present along the grain boundaries. As observed in Figure 1b–d, the introduction of the element La results in a reduction in the secondary dendrite arms of the grains, accompanied by an increase in the number of equiaxed grains. Moreover, as the La content increases, the grain size continuously decreases, with the most significant refinement observed in the 0.4 wt.% La alloy. However, with the further addition of La up to 0.6 wt.% La, a trend of coarsening and enlargement of microstructures becomes apparent, accompanied by irregular shapes and sizes of the grains. According to statistical analysis, the base alloy without La exhibits an average grain size of 50 μm, which decreases to 43, 26, and 27 μm after the addition of La. Therefore, the addition of La can significantly refine the grains in the alloy.

### 3.2. SEM Observation

**Figure 2:**
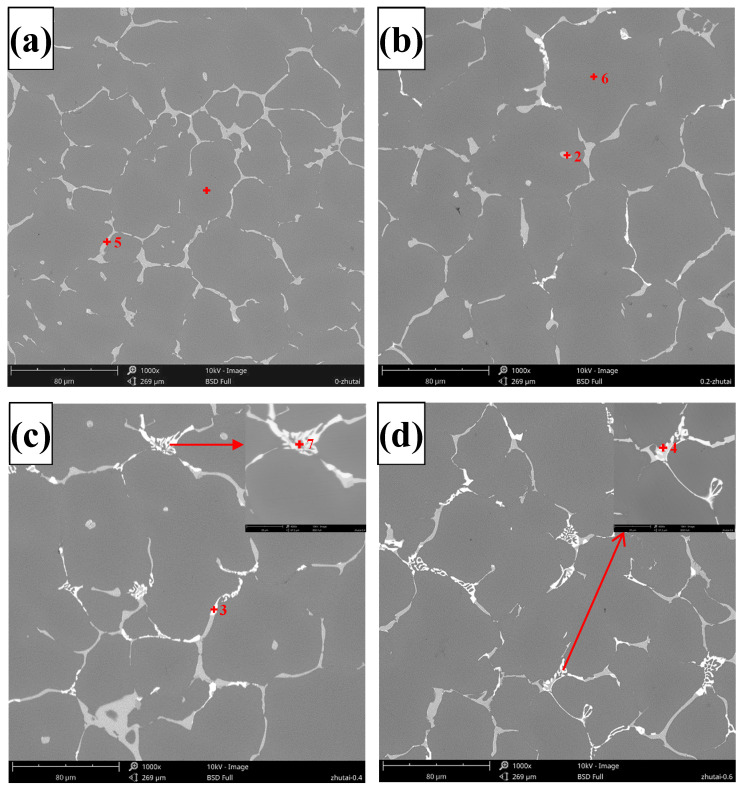
Microstructure of the Al-5.4Cu-0.7Mg-0.6Ag-xLa alloys: (**a**) La-free; (**b**) 0.2La; (**c**) 0.4La; (**d**) 0.6La.

**Figure 3:**
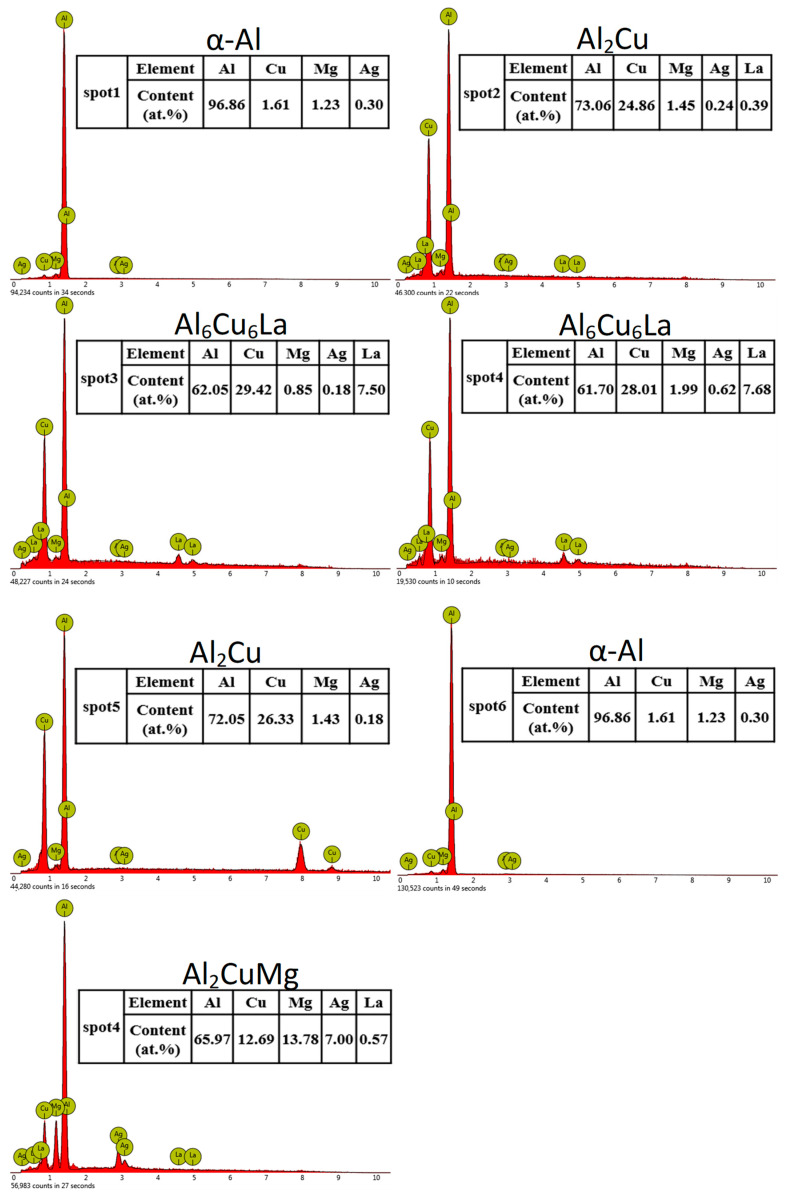
EDS composition analysis for each point listed in Table 3.

Figure 2 and Figure 3 depict the SEM images and corresponding EDS spectra of the Al-5.4Cu-0.7Mg-0.6Ag-xLa alloys, respectively. Table 3 presents the EDS spectrum analysis results of the Al-5.4Cu-0.7Mg-0.6Ag-xLa alloys. From Figure 2, it can be observed that the alloy’s microstructure is primarily composed of a dark gray α-Al matrix accompanied by numerous reticular second phases. Combining Figure 3 with Table 3, the plate-like or granular material in Figure 2 is speculated to be Al_2_Cu, while the irregular gray phase precipitated along the edge or in the middle of Al_2_Cu may be Al_2_CuMg. The bright white phase precipitated on Al_2_Cu is possibly Al_6_Cu_6_La, with a minor dissolution of Mg and Ag. In the alloy, many undissolved second phases aggregate at grain boundaries, and the introduction of La minimally influences the refinement of these boundaries. However, the continuous addition of La alters the morphology and distribution of the second phase in the alloy. When the La content is equal to or greater than 0.4La, the shape gradually transforms from irregular plate-like to discontinuous skeleton-like. Moreover, the higher the La content is, the more obvious the segregation phenomenon of the Al_6_Cu_6_La phase is, and the skeleton-like structure in the alloy is also increased.

Based on the Darken–Gurry theory, the interaction strength “W” between alloying elements can be calculated based on their atomic radii and electronegativities. With increasing “W” value, the interaction between alloying elements strengthens, leading to a stronger tendency for compound formation. This can serve as a qualitative method to characterize the tendency for alloy compound formation. The interaction strength “W” can be expressed as Equation (1):

(1) $${W=[(R}_{A}-R_{B})]/(0.15\cdot R_{A}){]}^{2}{+[(N}_{A}-N_{B})/0{.4]}^{2}$$

And when rare earth elements are added, it can be formulated as Equation (2):

(2) $$W_{\left(\mathrm{Al}-X\right)-\mathrm{RE}}{=W}_{\mathrm{Al}-X}{+W}_{X-\mathrm{RE}}+8.8$$

where $R_{A}$ and $R_{B}$ denote the atomic radii of atoms A and B, and $N_{A}$ and $N_{B}$ denote the electronegativity of the A and B atoms.

According to Equation (1) and Table 4, W_Al-Cu_ = 1.01 and W_Al-Mg_ = 1.19. Substituting these values into Equation (2), we obtain W_(Al-Cu)-La_ = 5.78 and W_(Al-Mg)-La_ = −7.22. By comparing the results from both equations, it can be concluded that the addition of the element La greatly increases the interaction between Al and Cu elements and decreases the interaction between Al and Mg elements. Consequently, the appearance of the Al_6_Cu_6_La phase in the alloy is initiated, while the formation of the Al_2_CuMg phase is reduced [13]. When a significant amount of the Al_6_Cu_6_La phase is formed, it consumes a considerable amount of Cu elements, which greatly affects the formation of other excess second phases in the alloy, consistent with the observations in Figure 2d.

### 3.3. XRD Patterns

**Figure 4:**
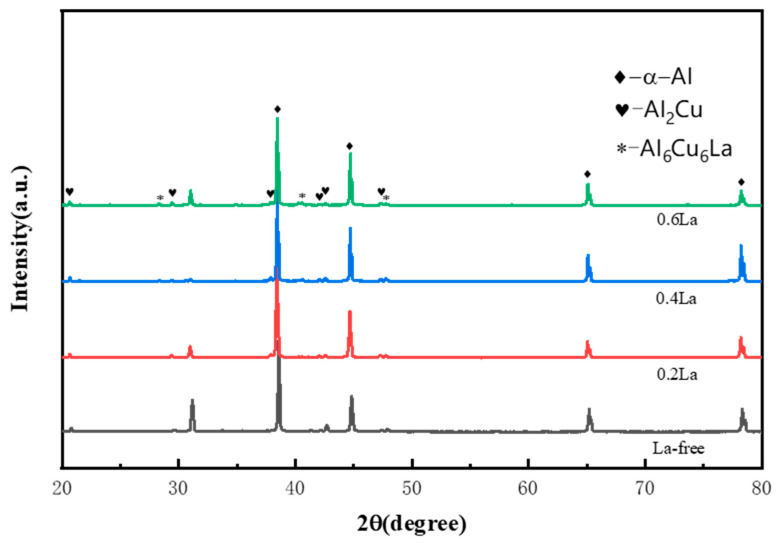
X-ray diffraction patterns of the Al-5.4Cu-0.7Mg-0.6Ag-xLa alloys.

The XRD spectrum of the Al-5.4Cu-0.7Mg-0.6Ag-xLa alloy is shown in Figure 4. From Figure 4, it can be observed that the diffraction pattern of the alloy exhibits strong characteristic peaks of α-Al and Al_2_Cu phases. It is evident that even after the addition of La, the primary phases of the alloy remain α-Al and Al_2_Cu. Furthermore, upon observation of the spectrum, it can be noted that with the addition of La, the diffraction peaks of the Al_2_Cu phase begin to weaken, primarily due to the reduction in the quantity of eutectic Al_2_Cu phase at the grain boundaries after La addition (as shown in fig4). Additionally, in the La-added alloy, the presence of the Al_6_Cu_6_La phase is observed. As a newly formed phase, Al_6_Cu_6_La not only affects the mechanical properties of the alloy at room temperature but also influences the mechanical properties of the alloy at high temperatures due to its thermal stability [13].

### 3.4. Tensile Properties

**Figure 5:**
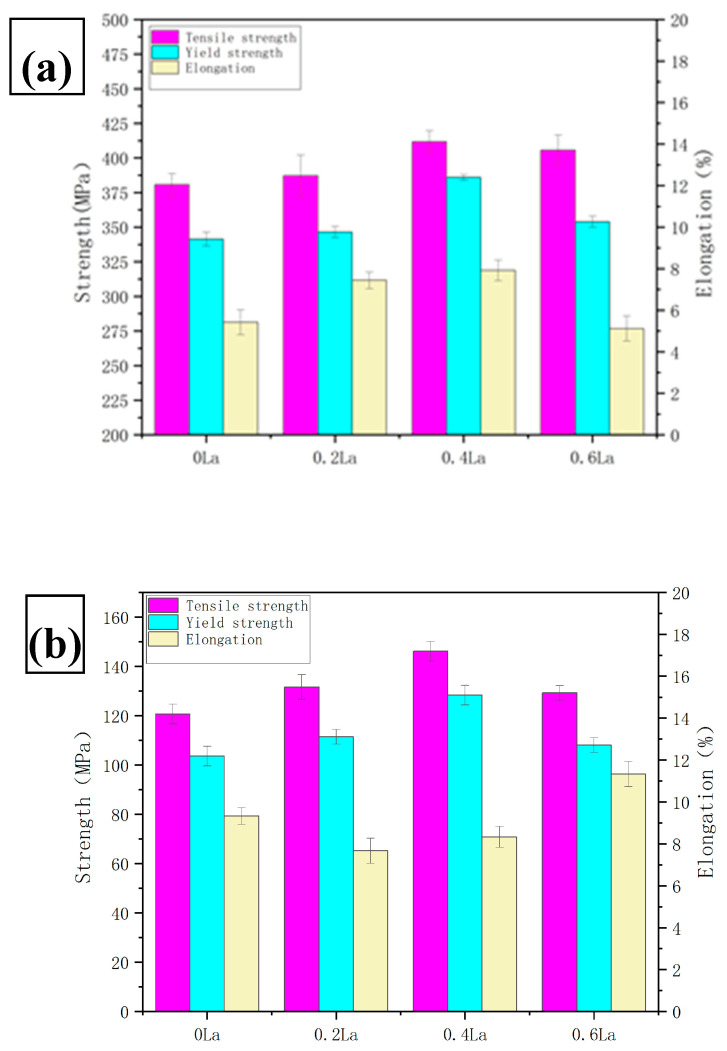
Mechanical properties of the alloys in the T6 condition at (**a**) room temperature, and (**b**) 350 °C.

The tensile mechanical properties of the alloys with varying La contents at room temperature are illustrated in Figure 5a and Table 5. The addition of La exerts a certain degree of influence on the properties of the Al-Cu-Mg-Ag alloy, but overall, the changes are not significant. The alloy containing 0.4 wt.% La demonstrates the highest tensile and yield strengths, reaching 411.7 MPa and 386.0 MPa, respectively. In comparison, the tensile and yield strengths of the alloy without La addition are slightly lower, at 380.6 MPa and 341.3 MPa, respectively. Alloys with 0.2 wt.% La and 0.6 wt.% La exhibit only minor improvements in tensile and yield strengths relative to the base alloy. The post-break elongation at room temperature follows a similar trend to the tensile strength, with the optimum value of 7.9% occurring in the alloy with 0.4 wt.% La.

The tensile mechanical properties of alloys with different La contents at 350 °C are depicted in Figure 5b and Table 6. It can be noted that the three types of heat-treated alloys exhibit a higher tensile strength at 350 °C compared to the untreated base alloy. Moreover, the high-temperature tensile strength and yield strength of the alloy initially increase and then decrease with the continuous addition of La content. The alloy containing 0.4 wt.% La achieves the highest tensile strength and yield strength, measuring 146.1 MPa and 128.3 MPa, respectively. Compared to the base alloy, these values represent increases of 25.4 MPa and 24.7 MPa, respectively. The high-temperature elongation of the alloy decreases at first and then increases, and the elongation of the alloy added 0.6wt.%La increases by 2% compared with the matrix alloy without La. The Al-5.4Cu-0.7Mg-0.6Ag-xLa alloy demonstrates considerable strength at room temperature (411.7 MPa) and 350 °C (146.1 MPa). The room temperature tensile strength of the alloy is superior to that of the Al-4Cu-1Mn alloy with the addition of 0.2CeLa and 0.1GdY, which is 260.9 MPa [18], as well as the Al-Si-Cu alloy with the synergistic effect of La and Y, which is 215.3 MPa [19]. At a high temperature of 350 °C, the tensile strength of the alloy is also significantly better than that of the Al-12.95Si-3.57Cu-0.72Mg-0.91Ni-0.53Fe-0.4Er alloy, which is 117 MPa [20] (as shown in Table 7), and the alloy strength is superior to that of many heat-resistant alloys. This means that adding a certain amount of lanthanum to the aluminum alloy can enhance the high-temperature tensile strength of the aluminum alloy.

Research has shown that the yield strength of metallic materials is correlated with grain size, as described by the Hall–Petch equation (as shown in Equation (3)) [21]:

(3) $$\sigma_{s}=\sigma_{0}+kd^{-\frac{1}{2}}$$

where *σ_s_* represents the yield stress of the specimen, *σ*_0_ is a constant associated with the initial stress required for dislocation motion, *k* is the strengthening coefficient, and *d* denotes the average grain diameter. As indicated by Equation (3), the addition of La leads to grain refinement in the alloy, and the smaller the grain size is, the higher the material’s yield strength is. This is consistent with the experimental results mentioned above.

**Table 5:** Data on the mechanical properties for the heat-treated Al-5.4Cu-0.7Mg-0.6Ag-xLa alloys evaluated at room temperature.

Alloys	UTS($\sigma_{b}$)/MPa	YS($\sigma_{s}$)/MPa	El./%
La-free	380.6 ± 8	341.3 ± 5	5.4 ± 0.6
0.2La	387.0 ± 15	346.4 ± 4	7.4 ± 0.4
0.4La	411.7 ± 8	386.0 ± 2	7.9 ± 0.5
0.6La	405.7 ± 11	353.9 ± 4	5.1 ± 0.6

**Table 6:** Data on the mechanical properties for the heat-treated Al-5.4Cu-0.7Mg-0.6Ag-xLa alloys evaluated at 350 °C.

Alloys	UTS($\sigma_{b}$)/MPa	YS($\sigma_{s}$)/MPa	El./%
La-free	120.6 ± 4	103.5 ± 4	9.3 ± 0.4
0.2La	131.6 ± 5	111.4 ± 3	7.6 ± 0.6
0.4La	146.1 ± 4	128.3 ± 4	8.3 ± 0.5
0.6La	129.1 ± 3	108.0 ± 3	11.3 ± 0.6

**Table 7:** Comparison of tensile strength of different heat-resistant aluminum alloys at 350 °C.

Materials Composition (wt.%)	Temperature (°C)	UTS($\sigma_{b}$) (MPa)	References
**Al-5.4Cu-0.7Mg-0.6Ag** **Al-12.95Si-3.57Cu-0.72Mg-0.91Ni-0.53Fe-0.4Er**	350 350	146.1 117	Present work [20]
**Al-12Si-3Cu-1.5Ni**	350	≈62	[22]
**Al-12Si-4Cu-2Ni-1Mg-AlNp**	350	106	[23]
**Al-6Cu-0.4Mn-0.4Ag**	350	135.8	[24]
**Al-11.79Si-3.33Cu-0.172Fe-2.05Mn-1Cr**	350	106	[25]
**A1-0.4Cu-8.2AlN**	350	119	[26]
**Al-12Si-0.9Cu-0.8Mg-4Ni**	350	116	[26]
**Al-12.5Si-0.84Mg-5Cu-2Ni-0.5Fe-(0.24~0.28) Cr**	350	≈92	[27]
**Al-12.87Si-5.45Cu-1.83Ni**	350	93.5	[28]
**Al-13Si-5Cu-0.6Fe-0.6Mn**	340	97	[29]
**Al-12.8Si-3.23Cu-1.01Mg-1Ni**	350	61.7	[30]
**Al-12.57Si-1.02Cu-1.23Mg-1.07Ni-0.15Mn**	350	75.6	[31]

### 3.5. High-Temperature Fracture

**Figure 6:**
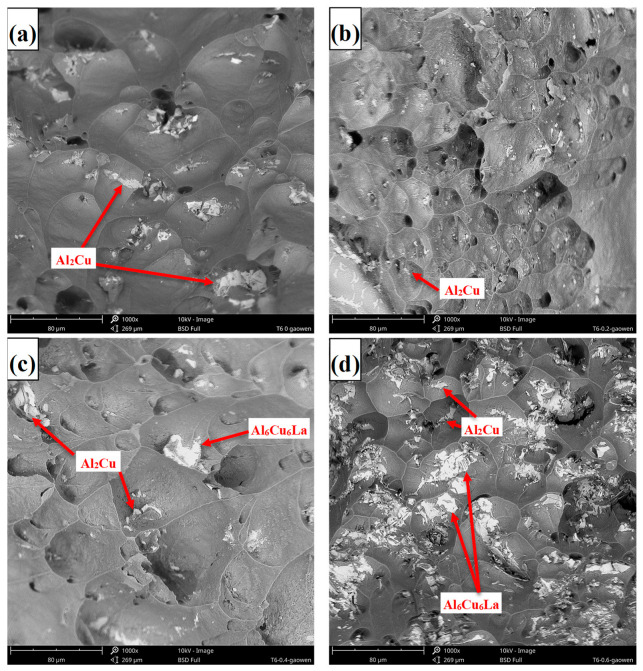
Tensile fracture morphology of the heat-treated Al-5.4Cu-0.7Mg-0.6Ag-xLa alloys: (**a**) La-free; (**b**) 0.2La; (**c**) 0.4La; (**d**) 0.6La.

The morphologies of tensile fractures at high temperatures for the Al-5.4Cu-0.7Mg-0.6Ag-xLa alloys are illustrated in Figure 6. All four alloy compositions exhibit uniformly spaced dimples on the fracture surfaces, indicative of typical ductile fracture behavior. The formation of these dimples results from the aggregation of voids. From the images, in the alloy lacking La addition, the dimples on the fracture surface are comparatively larger and shallower. The observed presence of sizable undissolved Al_2_Cu blocks in the unmodified alloy is linked to its reduced tensile strength, suggesting that the Al_2_Cu phase maintains good thermal stability owing to the solid solution of Mg and Ag atoms. With increasing La content, the dimple size initially decreases before increasing again. Additionally, the second phase within the dimples gradually transforms from a fine dispersed Al_2_Cu phase to a fine fragmented Al_6_Cu_6_La phase. Regarding fracture behavior, the dimples of the alloy with 0.2 wt.% La added are smaller and progressively increase in size with further La addition, and the dimples are larger and deeper in the alloy with 0.6 wt.% La added. This outcome aligns with the elongation data presented in Figure 5, indicating that increasing La content can enhance the ductility of the alloy.

### 3.6. TEM Microstructures

**Figure 7:**
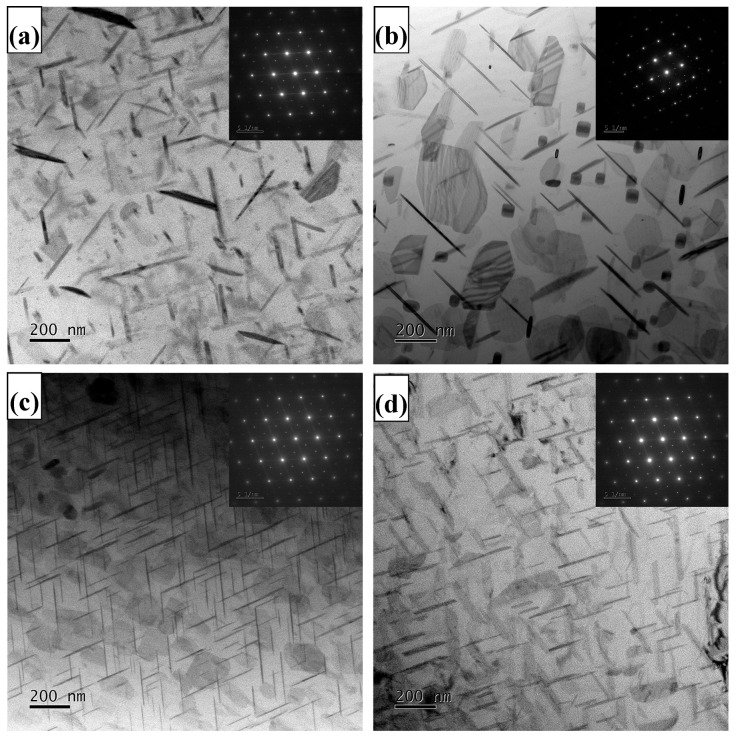
Bright-field TEM micrographs of the four T6-tempered Al-5.4Cu-0.7Mg-0.6Ag-xLa alloys taken close to <110> α zone axis (**a**) La-free; (**b**) 0.2La; (**c**) 0.4La; (**d**) 0.6La.

The TEM microstructure and corresponding diffraction patterns of the T6-treated Al-5.4Cu-0.7Mg-0.6Ag-xLa alloy are shown in Figure 7. Figure 7 includes bright-field TEM images and selected area electron diffraction (SAED) patterns from the <110>α orientation for the various alloy compositions. The SAED patterns reveal prominent diffraction spots at the 1/3 and 2/3 positions of {022}α, which confirm the presence of the Ω phase in all alloy compositions post-T6 treatment. The quantitative results for the number density and volume fraction of the Ω phase are provided in Table 8. Comparing Figure 7 with Table 8, it is apparent that the introduction of La leads to a reduction in the size of the Ω phase precipitates and an increase in their density.

**Figure 8:**
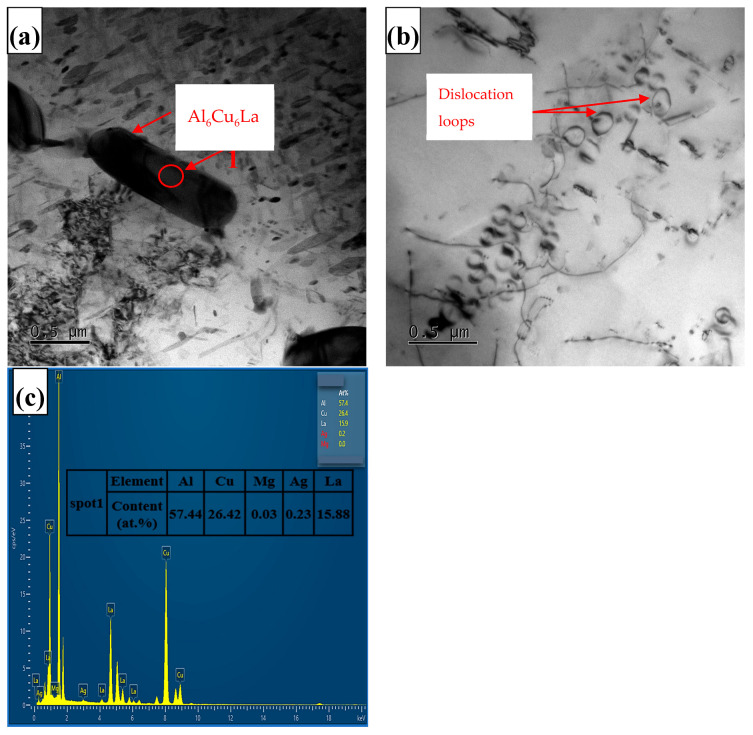
Microstructure at grain boundaries in La-modified alloys (**a**) and the dislocation relationship of the precipitated phase in the alloy (**b**) and the EDS analysis of point 1 (**c**).

The microstructure at the grain boundary and the dislocation relationship of the precipitated phase of the Al-5.4Cu-0.7Mg-0.6Ag-0.6La alloy are depicted in Figure 8. From Figure 8a and the energy spectrum corresponding to point 1 in Figure 8c, we can see that the Al_6_Cu_6_La phase exhibits a blocky morphology and enriches at the grain boundary. In Figure 8b, we observe a dislocation entanglement caused by the interaction of moving dislocations during the tensile process. According to the theory of combined dislocations, when external stress increases, dislocations are forced to move forward and bend around second-phase particles and other inclusions in the alloy, leaving dislocation loops. These products exert a repulsive force on the dislocation loops. The mutual accumulation of dislocations also causes them to be affected by pile-up stress, making the dislocation loops stable under the combined forces after the external force is removed.

## 4. Discussion

According to the microstructures of both unmodified and La-modified Al-5.4Cu-0.7Mg-0.6Ag alloys (as shown in Figure 1 and Figure 2), for the unmodified Al-5.4Cu-0.7Mg-0.6Ag alloy without La addition, the microstructure primarily consists of coarse α-Al dendrites and the Al_2_Cu eutectic phase. The primary α-Al dendrite cell size is around 50 μm, with coarse and unevenly sized dendrites. After La modification treatment with different compositions, the grain size is refined, with α-Al dendrite sizes ranging from 43 to 26 μm. The addition of rare earth La can significantly refine the grains of the Al-Cu-Mg-Ag alloy and effectively improve its microstructure, and the reference [32,33] has also confirmed the role of lanthanum in this refinement of alloy grains. This is mainly due to the emergence of the Al_6_Cu_6_La phase, which acts as a heterogeneous nucleation agent. In addition, the Mullins–Sekerka theory of interfacial stability dynamics defines the essential conditions for preserving interfacial stability, as outlined in Equation (4):

(4) $$G_{L}/v\;\geq {\;[m}_{L}C_{0}(1\;-{\;k)/\mathrm{kD}}_{L}]\cdot {[(k}_{S}{+k}_{L}{)/2k}_{L}]\;\cdot {\;\varphi +\rho }_{L}{H/2k}_{L}$$

where k can be expressed by the following Equation (5).

(5) $$k=C_{S}/C_{L}$$

where $G_{L\;}$ represents the temperature gradient at the solid–liquid interface front, v is the growth velocity of the solid–liquid interface, $m_{L}$ denotes the liquidus slope of the alloy, $C_{0}$ is the initial alloy composition, k is the solute partition coefficient, $D_{L\;}$ is the solute diffusion coefficient, $k_{S}$ and $k_{L}$ are the thermal conductivities of the solid and liquid phases, respectively, $\rho_{L}$ is the liquid phase density, H is the latent heat of fusion, φ  is the non-dimensional parameter, and $C_{S}$ and $C_{L}$ are the equilibrium solubilities.

When the addition of La is less than 0.4 wt.%, the solubility of La in Al is low, which hinders solute diffusion and reduces the $C_{S}$ value. Therefore, during the solidification process, La will be enriched in the front of the solid–liquid interface due to the redistribution of the solute, which increases the critical value of $G_{L}/v$ and increases the degree of subcooling of the components, which will increase the number of α-Al dendrites [34] and thus refine the α-Al dendrites. However, when the La content is greater than or equal to 0.4 wt%, the excess La forms intermetallic compounds, shown as Figure 3, resulting in a decrease in the $C_{S}$ and $C_{L}$ values, a decrease in the critical value of $G_{L}/v$, and the eventual coarser grain size [12].

Following the addition of La into the alloy, many bright white Al_6_Cu_6_La phases gradually precipitate at the edges or tails of the Al_2_Cu phase. The emergence of a limited quantity of the Al_6_Cu_6_La phase, due to its good thermal stability and selective precipitation in the local region, effectively promotes grain boundary strengthening, acting to immobilize grain boundaries and prevent grain boundary sliding [35]. Nevertheless, with the La content surpassing 0.4 wt.%, the finely granular Al_6_Cu_6_La phase gradually replaces the Al_2_Cu dendrites. Although it can refine the grains, it does not significantly enhance the alloy’s strength. This is because La primarily forms the Al6Cu6La phase within the alloy, which reduces the content of solid-soluble Cu atoms in the alloy and greatly reduces the formation of other excess second phases. Meanwhile, the pronounced interaction between La atoms and vacancy hinders the formation of Ag-Mg clusters, inhibits the nucleation of Ω phase, and delays the aging response of the alloy [36,37]. Additionally, in this investigation, the primary factors influencing the mechanical properties of Al-5.4Cu-0.7Mg-0.6Ag-xLa alloys include grain boundary strengthening, solid solution strengthening, and precipitation strengthening. As the grain size of the alloy continues to refine with the addition of La, the number of grains and grain boundaries increases. According to the Hall–Petch equation Formula (3), the addition of La increases the yield strength of the material, providing grain boundary strengthening to the alloy. The small amount of Al_6_Cu_6_La that forms segregates in local regions, also effectively promoting grain boundary strengthening. According to the calculations from Formulas (1) and (2), the addition of La greatly enhances the interaction between Al and Cu elements, resulting in more Al_2_Cu forming in the alloy. After solution treatment, the lower-melting-point phases Al_2_Cu and Al_2_CuMg dissolve into the α-Al matrix at a slower rate, making the eutectic phase discontinuous, causing lattice distortion in the matrix and resulting in solid solution strengthening. As shown in Figure 7 and Table 8, the addition of La affects the precipitation of the Ω phase, influencing the precipitation strengthening of the alloy. Therefore, the overall yield strength ($\sigma_{S}$) resulting from the interplay of these strengthening mechanisms can be represented by the following Formula (6) [38,39,40,41]:

(6) $$\sigma_{S\;}{=\sigma }_{\mathrm{gb}\;}{+\sigma }_{\mathrm{SS}}{+\sigma }_{\mathrm{ppt}}$$

where $\sigma_{\mathrm{gb}}$ denotes the increment in yield strength attributed to the effect of grain boundary strengthening. $\sigma_{\mathrm{SS}}$ represents the rise in yield strength resulting from solid solution strengthening, which is due to the dissolution of solute atoms into the matrix. $\sigma_{\mathrm{ppt}}$ indicates the enhancement of yield strength due to the presence of precipitates within the alloy.

Along the edge or in the middle of Al_2_Cu, irregular gray Al_2_CuMg phases precipitate as coarse residual secondary phases, acting as crack initiators, which reduce the ductility of the aluminum alloy [42]. From Table 3 and Figure 5, it can be observed that the rapid decrease in the elongation of the 0.2 wt.% La and 0.4 wt.% La alloys is related to this block-like secondary phase. Thus, the formation of Al_2_CuMg phases is identified as the cause of the mechanical performance decrease at room temperature.

As the concentration of La increases, a large amount of Al_6_Cu_6_La phases appear in the alloys with added La, and the amount gradually increases, and the phase does not disappear after solid solution treatment because of its high-temperature stability. When La is 0.6 wt.% La, more Al_6_Cu_6_La phases appear, leading to the emergence of numerous skeletal secondary phases in the alloy and greatly reducing the generation of Al_2_CuMg [13]. This alteration changes the shape and distribution of secondary phases within the alloy. Li [43] and Zhang [44] found that when the content of Y exceeded 0.15 wt.% in the Al-Cu-Mn alloy, the skeletal structure of Al_8_Cu_4_Y formed through the transformation of Al_2_Cu phase increased as the content of Y increased. In Figure 2, the abundant formation of Al_6_Cu_6_La phases in the alloy reduces the size of excess secondary phases on the grain boundaries, which has a great impact on the plasticity of the alloy [42,45]. This is because in the process of stretching, the enhancement in the fineness of the secondary phase in the alloy not only elevates the critical stress required for fracture at the grain boundaries but also weakens the internal stress between phases, reducing the initiation of cracks. Moreover, the refinement of the second phase makes it difficult for macroscopic shear bands to form in the alloy and cracks to extend outward [36,46,47]. Therefore, a large amount of Al_6_Cu_6_La phase increases the plasticity of the alloy.

According to the morphologies of the fracture in Figure 6, observations reveal that the size of the broken Al_2_Cu phase and the dimple in the fracture of the alloy modified by La decreases, and the second phase in the dimple is gradually replaced by the fine broken Al_6_Cu_6_La. The size of the dimple is determined by the decrease in the distance between undissolved secondary phases following grain refinement. Additionally, the depth of the dimples is governed by the extent of plastic deformation within the matrix. For the age-strengthened alloy with a small amount of La added, the Al_2_Cu particles dissolved in the matrix have a precipitation strengthening effect on the matrix due to the Orowan mechanism, and when the addition amount reaches 0.6 wt.% La, the large-sized Al_6_Cu_6_La will break and become fragmented in the high-temperature tensile strength at 350 °C, which results in a limited enhancement of the alloy’s strength and a decline in its mechanical properties.

The Ω phase, which serves as the primary strengthening phase within the alloy, is associated with the formation of Mg-Ag clusters resulting from the interaction between Mg and Ag atoms. These Mg-Ag clusters can function as nucleation centers for the Ω phase during the aging process, thereby facilitating its precipitation [48]. From Figure 7, we can observe that once the La concentration exceeds 0.4 wt.%, the size of the Ω phase rapidly increases, the number decreases, and the distance between the Ω phases becomes larger. This reduces the obstruction to dislocations, thereby reducing the effectiveness of precipitation hardening and a significant drop in alloy strength. This occurs because when the La concentration surpasses 0.4 wt.%, the substantial development of Al_6_Cu_6_La consumes some of the Cu, reducing the Cu available for the formation of the Ω phase, which also leads to a reduction in the quantity of Ω phases formed during the aging process [49]. The TEM images in Figure 7 confirm this viewpoint.

## 5. Conclusions

This study has investigated the influence of La on the microstructure and mechanical properties of Al-Cu-Mg-Ag alloys. The results show the following:

(1) La can refine the grain of the Al-5.4Cu-0.7Mg-0.6Ag alloy, but the refining effect begins to weaken when the content of La is 0.6wt%.
(2) At 350 °C, the tensile strength of the alloy increases with the increasing La content, and the elongation decreases first and then increases. The high-temperature tensile strength of the Al-5.4Cu-0.7Mg-0.6Ag alloy with added 0.4wt.% La at the T6 state is higher, which is 25.44MPa greater compared to the alloy without La.
(3) The La element mainly exists in the form of the Al_6_Cu_6_La phase in the Al-5.4Cu-0.7Mg-0.6Ag alloy, which makes the excessive second phase in the grain boundary appear skeleton-like; at the same time, the size of the excessive phase is reduced, and the high-temperature plasticity of the alloy is improved.
(4) In the La-added alloy, there exists an Al_6_Cu_6_La intermetallic compound distributed along the grain boundaries, which, after undergoing tensile deformation at 350 °C, breaks into fine particles, resulting in decreased mechanical properties.
(5) The addition of La refines the size of the strengthening precipitate phase Ω and increases its precipitation density.

## Figures and Tables

**Table 1:** Nominal compositions of Al-5.4Cu-0.7Mg-0.6Ag-xLa alloys for test (wt.%).

Alloy	Cu	Mg	Ag	La	Al
La-free	5.4	0.7	0.6	0	Bal.
2La	5.4	0.7	0.6	0.2	Bal.
4La	5.4	0.7	0.6	0.4	Bal.
6La	5.4	0.7	0.6	0.6	Bal.

**Table 2:** The actual composition of the experimental alloy (wt. %).

Alloy	Cu	Mg	Ag	La	Al
La-free	5.35	0.61	0.58	0	Bal.
2La	5.37	0.68	0.58	0.17	Bal.
4La	5.44	0.73	0.56	0.36	Bal.
6La	5.41	0.69	0.54	0.59	Bal.

**Table 3:** EDS analysis of the phases at the locations depicted in Figure 2 (at.%).

Spot	Al	Cu	Mg	Ag	La	Phase Type
1	96.86	1.61	1.23	0.30	0	α-Al
2	73.06	24.86	1.45	0.24	0.39	Al_2_Cu
3	62.05	29.42	0.85	0.18	7.50	Al_6_Cu_6_La
4	61.70	28.01	1.99	0.62	7.68	Al_6_Cu_6_La
5	72.05	26.33	1.43	0.18	0	Al_2_Cu
6	96.86	1.61	1.23	0.30	0	α-Al
7	65.97	12.69	13.78	7.00	0.57	Al_2_CuMg

**Table 4:** The atomic radii and electronegativities of the alloying elements [17].

Alloy Element	Al	Cu	Mg	La
Atomic radius (nm)	0.413	0.128	0.160	0.187
Electronegativity	1.610	1.900	1.310	0.110

**Table 8:** The results by quantitatively calculating Ω phases in studied alloys after being T6-tempered.

Alloys	Average Plate Diameter (nm)	Average Plate Thickness (nm)	Number Density (m^−3^)	Volume Fraction (%)
La-free	189.27	16.85	$9.70\times 10^{19}$	4.61
0.2La	221.07	16.70	$1.25\times 10^{20}$	8.01
0.4La	163.92	8.06	$6.97\times 10^{20}$	11.86
0.6La	152.31	17.66	$2.95\times 10^{20}$	9.51

## Data Availability

Data are contained within the article.
